# P-52. Modeling the Epidemiological Impact of Different Adult Vaccination Strategies in the United Kingdom

**DOI:** 10.1093/ofid/ofae631.259

**Published:** 2025-01-29

**Authors:** Rachel Oidtman, Giulio Meleleo, Ian Matthews, Dionysios Ntais, Robert Nachbar, Oluwaseun Sharomi, Kevin Bakker

**Affiliations:** Merck & Co., Inc., Lansdale, Pennsylvania; Wolfram Research, Inc., Champaign, Illinois; MSD (UK) Ltd., Value, Access, and Devolved Nations (VAD), London, England, United Kingdom; MSD (UK) Ltd., Value, Access, and Devolved Nations (VAD), London, England, United Kingdom; Wolfram Research, Inc., Champaign, Illinois; Merck & Co., Inc., Lansdale, Pennsylvania; Merck & Co., Inc., Rahway, NJ, USA, Philadelphia, Pennsylvania

## Abstract

**Background:**

Pneumococcal conjugate vaccines (PCVs) were first introduced in the pediatric United Kingdom (UK) immunization program in 2006 and subsequently led to a significant decline in invasive pneumococcal disease (IPD) for vaccine type serotypes (STs). Although pediatric PCVs provide some indirect protection to adults, a significant IPD burden remains in older adults. Here, we compared three adult vaccines in the 65+ year-old population: the current recommendation of PPV23, the recently licensed-in-adults PCV20, and a new adult-focused 21-valent PCV, V116. All scenarios assumed continued PCV13 vaccination in pediatrics.

**Methods:**

A population-level compartmental dynamic transmission model was adapted to the UK setting. The model described *Streptococcus pneumoniae* (SP) carriage transmission dynamics and disease progression in the presence of age- and ST-specific pneumococcal vaccines. We calibrated the model to age- and ST-specific IPD data in the UK. We used the calibrated model to make epidemiological projections under the three different vaccination scenarios.

**Results:**

After 10 years, preliminary results show the routine use of V116 or PCV20 in 65+ year-olds resulted in 17.7% and 13.9% reductions in IPD incidence from current values, respectively. The continued use of PPV23 resulted in a 0.6% increase in IPD incidence. Under the V116 vaccination scenario, we projected a 15.5% reduction in IPD incidence in STs included in V116 but not PCV20 (9N, 15A, 15C [generated from deOAc-15B], 16F, 17F, 20A, 23A, 23B, 24F, 31, and 35B) in 65+ year-olds, compared to 14.5% and 10.7% increases in incidence with PCV20 or PPV23 vaccination, respectively. For STs included in PCV20 but not V116 (1, 4, 5, 6B, 9V, 14, 15B 18C, 19F, and 23F), 10-year projections revealed decreases of 27.4%, 49.2%, and 32% in IPD incidence from the current incidence values with V116, PCV20, or PPV23 vaccination in the 65+ year-old population.

**Conclusion:**

Routine vaccination with V116 in older adults led to greater reductions in IPD among the 65+ year-old population compared to PCV20 and PPV23 vaccination. The model projected that with V116 vaccination in older adults, there would be no resurgence of the STs included in PCV20 but not V116 due to indirect protection offered by the pediatric vaccination program.

**Disclosures:**

**Rachel Oidtman, PhD**, Merck & Co., Inc.: Full time employee|Merck & Co., Inc.: Stocks/Bonds (Public Company) **Giulio Meleleo, PhD**, Merck & Co., Inc.: Vendor **Ian Matthews, PhD**, MSD (UK) Ltd., Value, Access, and Devolved Nations (VAD): Full time employee|MSD (UK) Ltd., Value, Access, and Devolved Nations (VAD): Stocks/Bonds (Public Company) **Dionysios Ntais, n/a**, MSD (UK) Ltd., Value, Access, and Devolved Nations (VAD): Full time employee|MSD (UK) Ltd., Value, Access, and Devolved Nations (VAD): Stocks/Bonds (Public Company) **Robert Nachbar, PhD**, Merck & Co., Inc.: US 7,219,020, US 5,292,741|Merck & Co., Inc.: Vendor|Merck & Co., Inc.: Stocks/Bonds (Public Company) **Oluwaseun Sharomi, MSc, PhD**, Merck & Co., Inc.: Full time employee|Merck & Co., Inc.: Stocks/Bonds (Public Company) **Kevin Bakker, PhD**, Merck & Co., Inc.: Grant/Research Support|Merck & Co., Inc.: Stocks/Bonds (Public Company)

